# Outcome of Early-Stage Glottic Laryngeal Carcinoma Patients Treated with Radical Radiotherapy Using Different Techniques

**DOI:** 10.1155/2019/8640549

**Published:** 2019-11-06

**Authors:** Oguz Cetinayak, Ersoy Dogan, Ahmet Kuru, Nesrin Akturk, Barbaros Aydin, Cenk Umay, Ilhami Er, Fadime Akman

**Affiliations:** ^1^Department of Radiation Oncology, Dokuz Eylul University Faculty of Medicine, Izmir, Turkey; ^2^Dokuz Eylul University Faculty of Medicine, Department of Otorhinolaryngology Head and Neck Surgery, Izmir, Turkey

## Abstract

**Purpose:**

The aim was to evaluate the treatment outcomes and prognostic characteristics of patients with early-stage glottic laryngeal carcinoma who underwent radical radiotherapy (RT) with different techniques.

**Patients and Methods:**

Radiotherapy was applied using the 2D conventional technique between 1991 and 2004 (130 patients), 3DCRT until 2014 (125 patients), and by VMAT until January 2017 (44 patients). Clinical T stages were 38 (12.7%) for Tis, 209 (69.9%) for T1, and 52 (17.4%) for T2. Radiotherapy technique and energy, anterior commissure involvement, and stage were analyzed as prognostic factors.

**Results:**

The median total dose was 66 (50–70) Gy, and median follow-up time was 72 (3–288) months; 5-year disease-specific survival (DSS) rates were 95.8%, 95.5%, and 88.6%, respectively, in Tis, T1, and T2 stages. In multivariate analyses, anterior commissure involvement was found significant for all survival and local control rates. The patients treated with VMAT technique had better local control and DSS rates. However, these results were not statistically significant.

**Conclusion:**

In early-stage laryngeal carcinomas, radical RT is a function sparing and effective treatment modality, regardless of treatment techniques.

## 1. Introduction

T1–T2 N0 glottic laryngeal carcinomas can be treated with transoral laser excision (LS), open partial laryngectomy (PL), or radiotherapy (RT) [1]. In comparison with transoral laser surgery and RT, a significant difference in disease control and voice quality especially in T1a patients has not been described [1–5]. Although the data in T1b cases are limited, local control rates are better with RT [1, 4, 6]. Radiotherapy provides better functional status compared with partial surgery due to the capability of normal tissue protection. Additionally, in a more disseminated disease like T1b, a better local control over LS can be achieved with RT [4, 6, 7]. Five years of local control rates are 85–94% for T1 glottic cancers and 70–85% for T2 with radical RT in the literature [5, 7–11]. Since a randomized trial from Japan demonstrated better local control rates with hypofractionated RT regimens, the use of such treatment modalities became more common [12]. However, the increase in local control rates provided by different fractionation regimens and new RT techniques has no impact on overall survival rates. According to SEER data, majority of the patients die due to secondary cancers or nonmalignant diseases like cerebrovascular attack [13, 14].

The implementation of new radiotherapy techniques such as carotid-sparing treatments has led to a decrease in nonmalignant deaths and is used more often in the treatment of early-stage laryngeal carcinoma patients [15]. In this study, we evaluated the treatment outcomes and the prognostic factors of patients with early-stage glottic laryngeal carcinoma.

## 2. Materials and Methods

### 2.1. Patients

In this study, 299 cases with Tis-T1-T2/N0 glottic laryngeal carcinoma, who underwent radical RT between July 1991 and January 2017 according to glottic laryngeal carcinoma protocol, were evaluated retrospectively. The median age of patients was 64 (27–89), and a clear majority of them were men (96%). The histopathological diagnosis was squamous cell carcinoma in 261 (87.3%) cases and carcinoma in situ in 38 (12.7%) cases (Table 1).

### 2.2. Diagnosis and Staging

Patients were assessed with detailed head and neck and systemic physical examination, whole blood count, and chest X-ray. The patients were staged with direct laryngoscopy and biopsy under anesthesia. In patients with anterior commissure involvement, the cartilage involvement was evaluated by computed tomography (CT). Glottic staging of the American Joint Committee on Cancer (AJCC) has been used for staging [10]. Clinical T stages were as follows: Tis in 38 (12.7%) cases, T1 in 209 (69.9%) cases (55.9% T1a, 14% T1b), and T2 in 52 (17.4%) cases. All of the lesions were located at the larynx and were N0 and M0.

### 2.3. Radiotherapy

**Figure 1:**
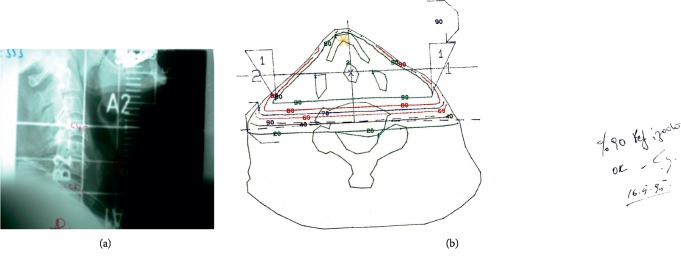
(a) 2D conventional radiotherapy fields for early-stage laryngeal carcinoma. (b) 2D CRT plan and isodose levels for early-stage Laryngeal carcinoma.

Radical RT was applied to patients with stage I glottic carcinoma and inoperable (low performance, comorbid disease, or patient preference) stage II disease. Patients were treated with two-dimensional (2D) conventional technique until 2004, with 3DCRT until 2014, and with VMAT later on. The patients were placed in supine position and fixed by using neck foam and orfit personal head-neck mask (in patients treated with 3DCRT and VMAT, by using shoulder-supported head and neck IMRT mask). RT planning was carried out as 2D (Figures 1(a) and 1(b)) with conventional simulation and by taking a single slice CT from the field center until the year 2004 and as 3D (3DCRT) by CT simulation after 2004.

Conventional and 3DCRT techniques have been applied with two parallel opposed fields using “wedge” and high-energy photons (Co60 or 6 MVX). A total of 66 Gy RT was applied in 33 fractions with 2 Gy/fraction, 5 fractions/week for Tis, T1 disease extending from the superior thyroid notch to the bottom of the cricoid cartilage and from 5–10 mm anterior to thyroid cartilage to the posterior of arythenoid cartilage. Whereas in stage II, the fraction scheme was applied with a total dose of 70 Gy in 35 fractions with the same field.

**Figure 2:**
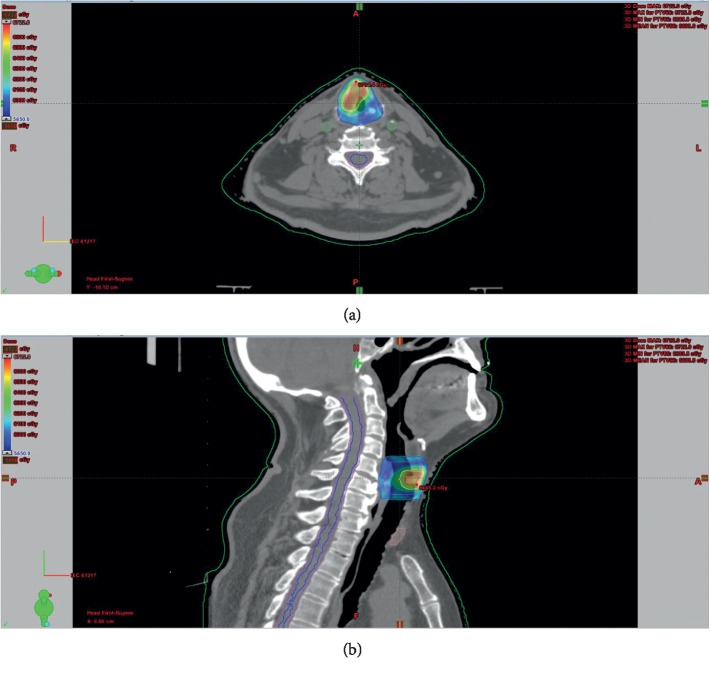
(a, b) VMAT planning for early-stage laryngeal carcinoma.

After 2014, patients were treated with the TrueBeam-STX machine, by using CT simulation with 1-2 mm slice thickness on the supine position and VMAT technique with 2 partial arcs, for carotid-sparing. The planning target volume (PTV1) was created by adding craniocaudal 5–10 mm, mediolateral, and anterioposterior 3–5 mm margin to GTV. The intermediate (60 Gy) clinical target volume (CTV) included the true vocal cord, ventricule, false vocal cords, arytenoids, aryepiglottic folds (for T2 disease), and subglottic region. PTV2 was created within the same margins as PTV1 to CTV. A total prescribed dose was 66–69 Gy to PTV1 and 60 Gy to PTV2, with simultaneous integrated boost (SIB) and IGRT technique in 30–33 fractions. In Tis-T1 tumors, RT was applied in 30 fractions to a total dose of 66 Gy and in T2 tumors 69 Gy in 33 fractions (Figures 2(a) and 2(b)).

### 2.4. Follow-Up

The treatment response and side effects were assessed at least once a week during RT. Response evaluation was done by flexible and/or direct laryngoscopy at 2–4 months after treatment. Follow-up was carried out every 2-3 months for the first 2 years after RT, every 6 months between 3 and 5 years, and once a year after 5 years.

Early and late side effects (according to RTOG/EORTC criteria) and the response assessment were carried out together with an ear, nose, and throat specialist and a radiation oncology specialist. Direct laryngoscopy and biopsy was performed in case of any pathologic findings. Annual chest X-ray was also performed. In any case of failure, each patient was evaluated individually and salvage treatments were planned by the Head and Neck Tumor Board.

### 2.5. Statistics

Overall survival was calculated from the first day of radiotherapy start to the any cause of death. The locoregional failure event/time was used to calculate disease-free survival. Disease-free survival (DFS) is defined as the time from radiotherapy start, until disease recurrence or death and endpoint for disease-specific survival (DSS) is death with laryngeal carcinoma. Stage, RT energy and technique, and anterior commissure involvement were evaluated as prognostic factors. Survival analysis was done by using SPSS v20.0 with the Kaplan–Meier method. Single and multivariate analyses were calculated by using Log-rank and Cox-regression tests with a 95% confidence limit for each survival analysis separately.

## 3. Results

One-hundred thirty (43.5%) patients were treated with the conventional technique, 125 (41.8%) cases were treated with 3DCRT, and 44 of them were treated with (14.7%) VMAT. The median dose of RT is 66 (50–70) Gy, and the median fractionated dose was 2 (1.8–3.12) Gy.

**Figure 3:**
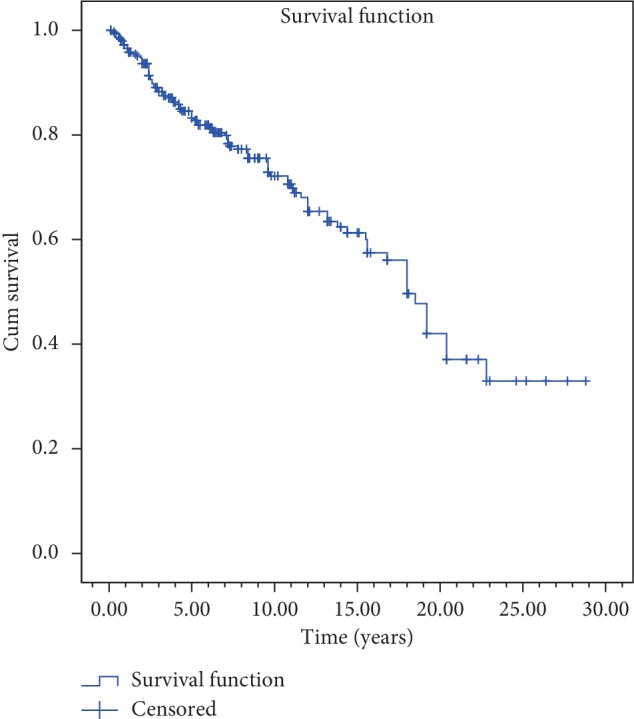
Overall survival curves.

**Figure 4:**
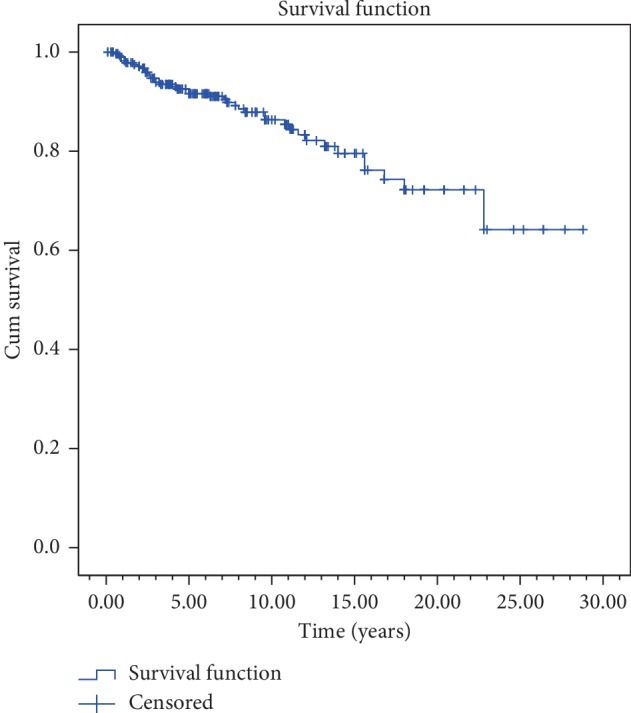
Disease-free survival curve.

Median follow-up was 72 (3–288) months. 2, 5, and 10 years of overall survival and disease-free survival rates were found to be 91.3%, 81.9%, and 65.4% and 87.6%, 72.3%, and 51.3%, respectively (Figures 3 and 4).

**Figure 5:**
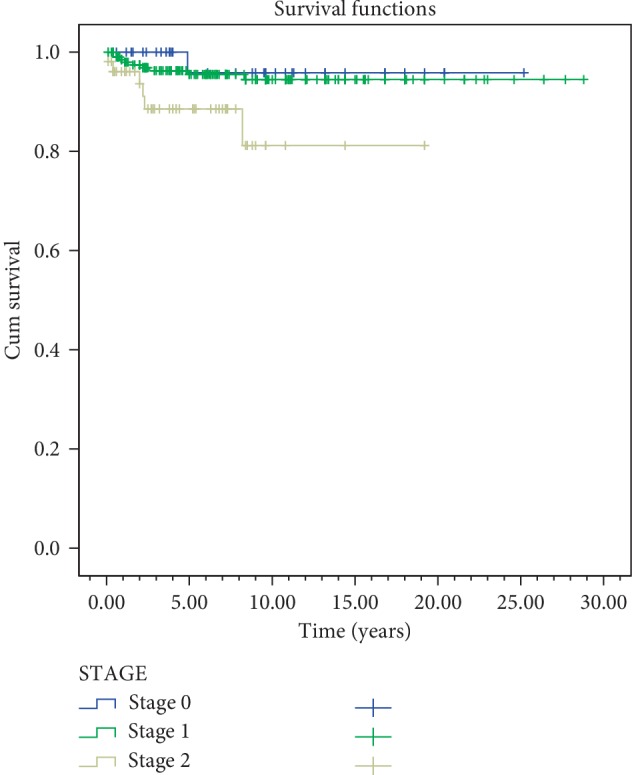
Disease-specific survival for clinical stage.

Disease-specific survival rates for 5 and 10 years according to stages were 95.8% and 95.8% for Tis; 95.5% and 94.5% for T1; and 88.6% and 81.2% for T2 diseases, respectively (Figure 5). Local control rates for 5 and 10 years are as follows: 79.2% and 79.2% for Tis; 93.1% and 92% for T1; and 78.7% and 66.6% for T2 were reached.

**Figure 6:**
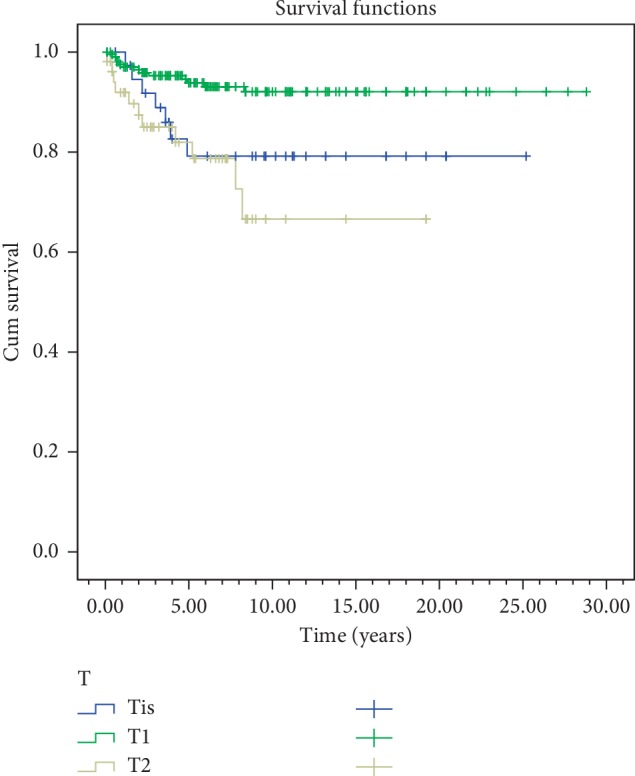
Local control by T classifications.

**Figure 7:**
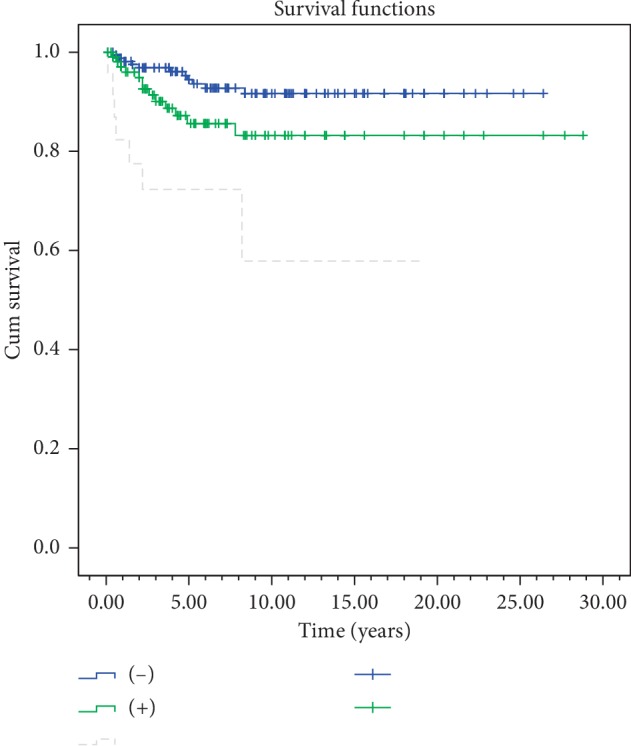
Local control by anterior commissure involvement.

**Figure 8:**
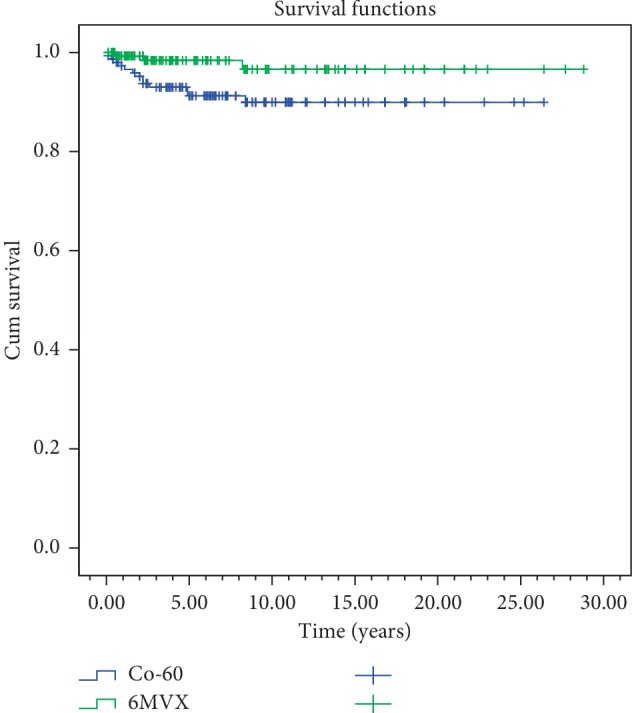
Disease-specific survival for beam energy.

**Figure 9:**
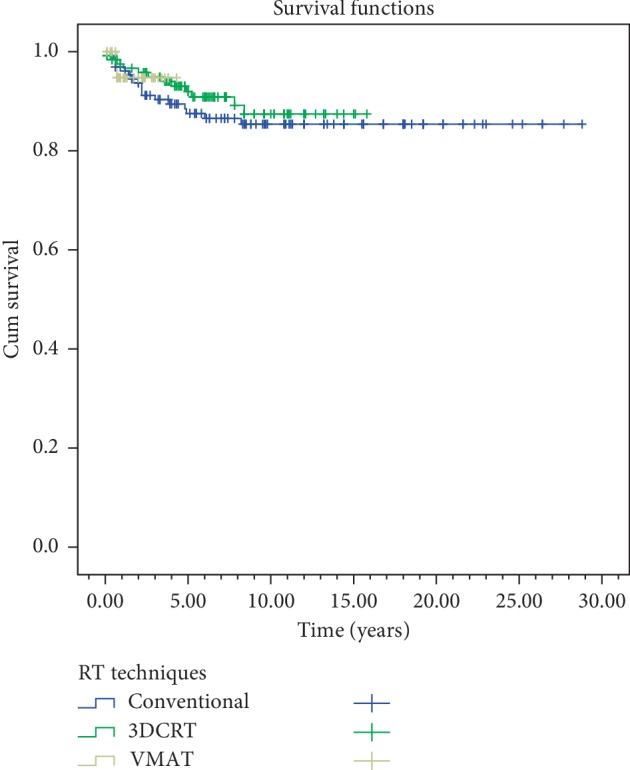
Local control curves by RT technique.

In the univariate analyses, stage, gender, anterior commissure involvement, RT technique, and RT energy were evaluated. In both overall survival and disease-free survival, stage (*p*=0.003) and anterior commissure involvement (*p* < 0.001) were found statistically significant. RT technique was not found to be significant in overall survival and disease-free survival (*p*=0.61,  *p*=0.51) (Table 2) (Figures 4–7). In disease-specific survival, stage (*p*=0.033), anterior commissure involvement (*p* < 0.001), and RT energy (6MV-X/Co60) (*p*=0.028) were found to be significant. In local control, stage (*p* < 0.001) and anterior commissure involvement (*p* < 0.001) were significant. However, the VMAT technique had better results in local control and disease-specific survivals. However, these results were statistically not significant (Table 2) (Figures 6–9).

In the multivariate analyses, anterior commissure involvement was found statistically significant in overall, disease-free, and disease-specific survival (*p* < 0.001, *p* < 0.001, *p* < 0.001). Only anterior commissure involvement (*p* < 0.001) was found statistically significant in local control (Table 3).

### 3.1. Local Failure and Salvage Treatments

Local failure was detected in 31 (10.36%) patients, and the median time to local failure was 22 (1–84) months. Regional failure was detected only in 5 (1.67%) patients, and the median time to regional failure was 40 (6–88) months. Distant failure developed in only one patient with T2 disease at 23^rd^ month as lung metastasis (Table 4). Salvage surgery was applied to 4 (3 partial and 1 total laryngectomy (PL, TL)) patients in Tis; 9 (3 PL and 6 TL) cases in T1; and 9 (3 PL and 6 TL) patients in T2. Five-year larynx preservation rates were calculated as 97.3% in Tis, 97.2% in T1a, 97.3% in T1b, and 86% in T2 after salvage treatments. In one patient (0.33%) with regional failure, neck dissection was performed followed by chemotherapy; 4 patients with regional failure (1.33%) were treated with RT and chemotherapy. Patients most frequently died due to nonmalignant (42 (14%)) reasons. Only 16 (5.4%) patients died due to laryngeal carcinoma, another 19 (6.4%) patients had died of lung cancer, and 13 (4.3%) of other malignancies (esophagus, bladder, and prostate).

### 3.2. Complications

RTOG grade 3 late side-effect was recorded only in 1 (0.3%) patient after 12 months from RT. That patient had T1 glottic larynx carcinoma and received 66 Gy/2 Gy fraction dose RT by the conventional technique and has continued to smoke during and after the treatment. Larynx was protected with conservative therapy. During the follow-ups, that patient died due to lung cancer in the 156^th^ month.

## 4. Discussion

Early-stage glottic laryngeal carcinomas can be cured by radical RT or local surgical excision [1]. The treatment decision depends on the patient's preference as well as the technical possibilities and experience of the treatment team or the disease-specific features such as tumor location, single or bilateral vocal cord, and anterior commissure involvement. When appropriate patient selection is made in tumor control, there is no difference between the methods. The voice quality is changed according to surgical methods, but RT generally has the advantage of providing better voice quality [2–6]. The leading properties are stage and anterior commissure involvement in the prognostic factors [9, 16–26].

In current study, 2, 5, and 10 years of overall survival and disease-free survival rates were found to be 91.3%, 81.9%, and 65.4% and 87.6%, 72.3%, and 51.3%, respectively (Figures 3 and 4). These results are similar to those reported in many studies. Johansen et al. from Denmark has evaluated 861 glottic laryngeal carcinoma cases retrospectively and reported 5 years of disease-specific survival rates for T1a, T1b, and T2 tumors to be 95%, 93%, and 83%, respectively [11]. Chera et al. evaluated retrospectively 585 patients with T1N0, T2N0 glottic larynx carcinoma treated by RT at Florida University. In this study, 5 years of local control rates were reported as 94% for T1a; 93% for T1b; 80% for T2a, and 70% for T2b [9]. Compared to these series from 1980 to 2010, the survival and control rates of our study are compatible or better. In these series, conventional fractionated RT was mostly applied. In 2006, Yamazaki et al. from Japan demonstrated that hypofractionated schemes provide higher local control and then short-term treatments have been increased to be used [7, 12, 16, 26–28]. Hypofractionated applications do not create a survival difference in every study but can create easiness in daily practice by shortening the total treatment time without any serious side effects. It is also important to note that there is a slight increase, especially in the early side effects despite the positive reflections of survival in hypofractionated applications [7, 28, 29]. However, this negativity can be ruled out by smaller treatment volumes and better planning and using image-guided radiotherapy techniques. In our protocol, however, the fraction doses above 2 Gy were started to be applied only by VMAT and IGRT techniques due to years of experience and very low side-effect ratio (0.3%). In patients treated with the new technique, better survival and local control rates were obtained although statistical significance was not found yet (Table 2).

In our study, stage, gender, anterior commissure involvement, RT energy, and RT technique are evaluated as prognostic factors in univariate analyses. Stage (*p* < 0.001) and anterior commissure involvement (*p* < 0.001) were found to be significant in overall survival and disease-free survival. These factors, which are related to the tumor location and spread characteristics, are most significant in the literature and are also important factors in treatment selection [19, 22, 24]. The clear majority of glottic cancers occurs in the anterior part of vocal cords and frequently invades anterior commissure. Anterior commissure directly holds on to thyroid cartilage without any perichondrial distinction, and this creates a weak area for the spread of the tumors. Therefore, worse local control in patients with anterior commissure involvement is expected. Anterior commissure involvement in our study was found to be an independent prognostic factor affecting local control and overall survival in multivariate analysis and is consistent with many literature data [19, 24, 26]. Improved planning techniques and the widespread use of IGRT routine practices can reduce the negative impact of anterior commissure involvement, especially in local control.

In this study, 5 years of local control rates in stage I and II cases were found to be 93.1% and 78.1%, respectively, and 79.2% in Tis. Different treatments such as laser surgery, vocal cord stripping, cordectomy, hemilaryngectomy, or radical radiotherapy can be applied in Tis patients [30, 31]. In the study of Nguyen et al., there was no significant difference in survival between vocal cord stripping and RT (87–100%) [32]. At the University of Florida, Tis patients achieved a 5-year local control rate of 88% with radical RT [33]. In 2010, the same group reached a local control of 91% with RT [30]. Lower local control of Tis cases in our study may be attributed to the fact that biopsy materials in laryngeal cancer may not reflect the entire tumor tissue. It is more appropriate to plan the treatment of these tumors according to clinical spread characteristics. In the Tis group, 7 local recurrences were detected and 4 of these 7 patients have anterior commissure involvement. We believe that due to the high anterior commissure involvement and most patients have been treated with old RT techniques, the local recurrence rate in the Tis group was close to the T2 group. Otherwise, the doses applied to the larynx were not different.

It has been known that factors like RT technique, RT energy, fractional dose, total dose, and treatment duration influence the success of radiotherapy in disease control [7, 23, 25, 27, 34]. In our study, stage (*p*=0.033) and anterior commissure involvement (*p* < 0.001) and RT energy (6MV-X/Co60, *p*=0.028) were found to be significant in the disease-specific survival (Table 2) (Figures 5–8). However, it should be kept in mind that this significance may not only be energy dependent but also the contribution of conformal and VMAT techniques in which 6 MV X is used. In this study, 5 years of local control and disease-free, disease-specific, and overall survival rates were 86.5%, 73.1%, 95.6%, and 81% for conventional RT technique; 90.9%, 71.8%, 92.2%, and 83% for 3D-conformal RT technique; and 94.7%, 76.7%, 100%, and 87.2% for VMAT technique, respectively (Table 2). But, in conclusion, RT technique was not found to be a significant factor in local control (*p*=0.769), disease-free (*p*=0.51), disease-specific (*p*=0.231), and overall (*p*=0.61) survivals (Table 2). To get more accurate results, more patients are needed to be treated with new techniques.

Gomez et al. compared 3DCRT with IMRT techniques; IMRT significantly reduced the dose of the carotid artery, while no significant difference was found in the target dose in both techniques. In the IMRT plan, the average dose given to the carotid artery was 2000 cGy lower when compared with 3DCRT. It has also been seen that it can be further decreased in plans preserving arytenoid with anteriorly located lesions [35]. Matthiesen et al. compared IMRT, Rapid Arc (RA), proton, and 3DCRT in the treatment of early-stage glottic laryngeal carcinoma. This study has shown that three techniques which are the new technologies have more homogenous dose distribution in PTV as well as a significant decrease in thyroid and carotid artery doses. In addition, RA is better than other new techniques in PTV dose and preservation of normal structures [36]. Samuels et al. have discussed the transition period to carotid-preserving IMRT techniques and the advantages in early-stage laryngeal carcinomas [15]. However, carotid-preserving treatments must also be carefully considered in terms of local failure of the tumor. Gujral DM et al. assessed 16 articles on this approach; they emphasized the need for consensus and prospective study for the definition of carotid preserving target volume in IMRT [37]. In this study, we aimed to preserve the carotid arteries by using the VMAT technique; but to make a significant difference in survival, long-term follow-up is needed.

Functional preservation of the larynx in the treatment of early-stage glottic laryngeal carcinomas is important to improve local control and survival. However, as with patients with laryngeal carcinoma in our series, the quality of life deteriorates frequently due to secondary malignancy or nonmalignant reasons and patients lose their lives [13, 16, 23]. Especially in patients above 65, there is no difference between surgery and RT, in terms of deaths caused by cardiac and cerebrovascular system diseases [14]. In the large group of patients in which risk factors have been evaluated by Al-Mamgani et al., death caused by laryngeal carcinoma is only 3%, but comorbidity (16%) and second primary tumor (8%) and deaths because of unknown reasons (8%) are higher [23]. Smoking is an important factor for deaths for other reasons. With new RT techniques, carotid preservation can reduce cerebrovascular events due to circulatory problems even though it does not affect death by cardiac causes [13].

Partial laryngectomy could be applied for 9 out of the 31 patients with local failure for salvation, but in 13 patients, total laryngectomy was necessary. Salvage surgery could not be applied to others because of either patient refusal or comorbidity. In the current study, according to the data in the literature, local failures are lower, and with the salvage treatments, higher local control and functional larynx rates have been achieved (95% 5 years) [9, 18]. However, it should not be forgotten that more larynx protective surgery can be applied with close follow-up and a higher quality of life with better function can be achieved [38].

## 5. Conclusion

As a result, in our series, it can be concluded that the patients with early-stage glottic laryngeal carcinoma, local control, overall, disease-free, and disease-specific survival rates that we have achieved with radical radiotherapy are compatible with the literature and side effects are less frequent. According to the results of randomized trials, a higher fractional dose can shorten the total treatment time and can be applied more safely with the help of current treatment techniques. By the early detection of failures with multidisciplinary follow-ups, effective voice protective salvage treatments can be applied. The use of carotid preservation techniques with the support of technology, better visualization of the anterior commissure, and more widespread application of plans such as VMAT and IGRT methods, high local controls and overall survival can be achieved.

## Figures and Tables

**Table 1:** Patients and treatment characteristics (*n* *=* 299).

Characteristics	*N*	%
Sex		
Male	287	96
Female	12	4
T Stage		
Tis	38	12, 7
T1a	167	55, 9
T1b	42	14
T2	52	17, 4
Anterior commissure invasion		
No	168	56, 2
Tis	30	10, 0
T1a	122	40, 8
T1b	12	4, 1
T2	4	1, 3
Yes	107	35, 8
Tis	8	2, 7
T1a	42	14, 0
T1b	30	10, 1
T2	27	9, 0
Unknown	24	8
Tis	0	0
T1a	3	0, 1
T1b	0	0
T2	21	7, 9
Fractionation		
1.8–2 Gy/fx, daily	297	99
>2 Gy/fx daily	2	1
Beam energy		
Cobalt-60	150	50, 2
(i) Conventional	52	17, 4
(ii) 3D-CRT	98	32, 8
6 MV X-rays	149	49, 8
Radiotherapy techniques		
Conventional	130	43, 5
3DCRT	125	41, 8
VMAT	44	14, 7

**Table 2:** Univariate analyses: prognostic factors for 5 year overall survival, disease-free survival, disease-specific survival, and local control.

Prognostic factor	Overall survival		Disease-free survival		Disease-specific survival		Local control	
	5 year (%)	*p*	5 year (%)	*p*	5 year (%)	*p*	5 year (%)	*p*
Stage		0.001		0.003		0.033		0.001
Stage 0	86		72.1		95.8		79.2	
Stage 1	84		74.9		95.5		93.1	
Stage 2	67.6		62.4		88.6		78.7	

Anterior commissure infiltration		<0.001		<0.001		<0.001		<0.001
No	87.3		79.3		96.7		92.7	
Yes	79		66.6		93.6		85.6	

Radiotherapy techniques		0.61		0.51		0.231		0.769
Conventional	81		73.1		95.6		86.5	
3DCRT	83		71.8		92.2		90.9	
VMAT	87.2		76.7		100		94.7	

Beam energy		0.52		0.55		0.028		0.266
Cobalt-60	79.2		72.2		91.2		86.6	
6 MV X-rays	85.4		72.5		98.4		91.4	
Log-rank test (95% CI)								

**Table 3:** Multivariate analysis for overall survival, disease-specific survival, disease-free survival, and local control.

	Overall survival	Disease-free survival	Disease-specific survival	Local control
Stage	NS	NS	NS	NS
Anterior commissure infiltrations	<0.001	0.001	0.01	<0.001
Radiotherapy techniques	NS	NS	NS	NS
Beam energy	NS	NS	NS	NS

NS: not significant.

**Table 4:** Recurrence rates.

Stage/(*n*)	Local recurrence	Regional recurrence	Distant metastases
Tis (38)	7 (18.4%)	—	—
T1 (209)	13 (6.2%)	4 (1.9%)	—
T1a (167)	10 (5.9%)	4 (2.4%)	—
T1b (42)	3 (7.1%)	—	—
T2 (52)	11 (21.1%)	1 (1.9%)	1 (1.9%)

## Data Availability

The patients data used to support the findings of this study are available from the corresponding author upon request.
